# Determinants of alcohol consumption and marijuana use among young adults in the Republic of Palau

**DOI:** 10.1186/s12199-020-00928-8

**Published:** 2021-01-22

**Authors:** Mizuki Sata, Renzhe Cui, Chifa Chiang, Singeru Travis Singeo, Berry Moon Watson, Hiroshi Yatsuya, Kaori Honjo, Takashi Mita, Everlynn Joy Temengil, Sherilynn Madraisau, Kazumasa Yamagishi, Atsuko Aoyama, Hiroyasu Iso

**Affiliations:** 1 Public Health, Department of Social Medicine, Osaka University Graduate School of Medicine, 2-2 Yamadaoka, Suita, Osaka, 565-0871 Japan; 2 Department of Preventive Medicine and Public Health, Keio University School of Medicine, 35 Shinanomachi, Shinjuku-ku, Tokyo, 160-8582 Japan; 3 Department of Public Health and Health Systems, Nagoya University School of Medicine, 65 Tsurumai-cho, Showa-ku, Nagoya, Aichi 466-8550 Japan; 4 Ministry of Health, Republic of Palau, One Hospital Road, P.O. Box 6027, Koror, 96940 Republic of Palau; 5 Department of Public Health, Fujita Health University School of Medicine, 1-98 Dengakugakubo, Kutsukake-cho, Toyoake, Aichi 470-1192 Japan; 6 Faculty of Medicine, Social and Behavior Sciences, Osaka Medical College, 2-7 Daigakumachi, Takatsuki, Osaka, 569-8686 Japan; 7 Faculty of International Relations, Kyoto Sangyo University, Motoyama, Kamigamo, Kita-ku, Kyoto, Kyoto 603-8555 Japan; 8 Department of Public Health Medicine, Faculty of Medicine, and Health Services Research and Development Center, University of Tsukuba, 1-1-1 Tennodai, Tsukuba, Ibaraki, 305-8575 Japan

**Keywords:** Non-communicable disease, Alcohol, Drug, Palau, Pacific islanders

## Abstract

**Background:**

This study aimed to describe the status of alcohol consumption and drug use among young adults as well as their determinants.

**Methods:**

We conducted a cross-sectional study of 356 young adults (aged 18 to 24 years) living in Palau in 2013. The prevalence of self-reported alcohol and marijuana usage were compared within and between sexes, age groups, ethnicities, and education levels.

**Results:**

The proportion of current drinking was higher in people aged 21–24 than in those aged 18–20 (73.2% vs. 60.9%, *p* = 0.09 in men and 48.3% vs. 30.0%, *p* = 0.02 in women), while that of marijuana use did not differ between the age groups. The proportions of current drinking and marijuana use were higher in Palauan than in other ethnicities (current drinking: 70.6% vs. 40.6%, *p* = 0.005 in men and 38.8% vs. 16.6%, *p* = 0.04 in women; lifetime marijuana use: 80.0% vs. 52.9%, *p* = 0.02 in men and 56.1% vs. 30.6%, *p* = 0.09 in women). The proportion of frequent (3 times or more) marijuana users was higher for the lower educated than for the higher educated (62.5% vs. 32.1%, *p* < 0.001 in men and 33.9% vs. 24.4%, *p* = 0.12 in women).

**Conclusions:**

Sex, age, ethnicity, and education were significant determinants of alcohol and marijuana use.

## Background

In 2010, the World Health Organization (WHO) published a global strategy to reduce the harmful use of alcohol which is a significant contributor to the global burden of disease and is listed as the third leading risk factor for premature deaths and disabilities in the world [1]. It is estimated that 3.3 million people worldwide died of alcohol-related causes, corresponding to 5.9% of all deaths in 2012 [2]. With regard to patterns of alcohol consumption, monthly heavy episodic drinking was slightly more prevalent among young people between 15 and 19 years of age (11.7%) than among the total population aged 15 years or older (7.5%) [2].

As for the most serious outcome that can result from illicit drug use, it is estimated that 187,100 drug-related deaths occurred worldwide (40.8% deaths per million aged 15–64) in 2013 [3]. In addition, 27 million people aged 15–64 are problematic drug users [3].

The Republic of Palau, an island state in Oceania with a population of approximately 20,000, has been reported to have a high burden of non-communicable diseases (NCDs) attributable to lifestyles including alcohol consumption or drug use, as with another countries in Micronesia [4]. In general, a meta-analysis that included nine studies (USA, UK, New Zealand, Finland, and Italy) showed that lower socioeconomic status was associated with higher prevalence of alcohol and marijuana use among adolescents aged 10–15 years [5]. The Youth Risk Behavior Surveillance System (YRBSS) found that 66.2% of Palauan high school students have previously consumed alcohol, 37.4% were currently consuming alcohol, 66.3% have previously used marijuana, and 38.4% were currently using marijuana [6]. However, whether student habits become worse or better after graduation remains unclear. Therefore, in this study, our aim was to investigate the prevalence of alcohol and marijuana use and their social and demographic determinants among young adults living in Palau.

## Methods

### Study subjects

This is a cross-sectional study involving the youth population from ages 18 to 24 years living in Palau. In October 2013, we established a survey station at Palau Community College (PCC), located in the center of Koror, since PCC is the only college-level education institution in Palau, provides optimum geographic access, and is the sole organization with a majority of members in the target age group (473 students). In order to reach as many potential participants as possible, we also dispatched a mobile survey team to a few local communities and major employers in Koror. A total of 356 people (310 Palauan and 46 non-Palauan) voluntarily participated in the survey. After excluding volunteers with missing answers for drug use (*n* = 1), a 17-year-old participant (*n* = 1), and pregnant women (*n* = 2), data from the remaining 352 participants (174 men, 178 women) were analyzed. None of the analyzed surveys had missing answers with regards to alcohol consumption.

The study protocol was reviewed and approved by the Osaka University Research Committee (approval number: 12145), the Nagoya University School of Medicine Bioethics Review Committee (approval number: 2012–0103), and the Institutional Review Board of the Ministry of Health, Republic of Palau. Written, informed consent was obtained from all of the participants prior to the study.

### Measurement and classification of variables

The questionnaire administered to participants consisted of lifestyle questions such as tobacco use, alcohol consumption, diet, physical inactivity, education level, household income, mental health, sleep habits, and illicit drug use. The details of the protocol used in this study are described elsewhere [7]. In brief, the questionnaire asked about alcohol consumption (ever consumed, consumed during the past 12 months, frequency of alcohol consumption during the past 12 months, and consumed during the past 30 days), marijuana use (past usage, age at the first use, and use during the past 30 days), ethnicity (Palauan, Filipino, or Other), and education (less than primary, primary completed, secondary school completed, and college/university completed).

### Statistical analysis

Calculation of the Kappa coefficient was used to assess correlations between current drinking (yes/no) and marijuana usage (yes/no) during the past month prior to the survey. Differences in proportions of alcohol and/or marijuana usage with respect to sex (men vs. women) and age groups (18 to 20 years old vs. 21 [legal drinking age in Palau] to 24 years old) were tested by analysis of variance (ANOVA). Sex-specific, and age-adjusted proportions for each outcome according to ethnicities (Palauan vs. others) and education levels (low: secondary school completed or less vs. high: college/university completed or more) were examined using analysis of covariance (ANCOVA). Differences in the distributions of the frequency of alcohol consumption during the 12 months prior to surveying and marijuana use between sexes and two age groups were analyzed by Chi-squared testing. Mantel-Haenszel tests were used for differences in the age-adjusted distributions of ethnicities and education levels.

All statistical analyses were performed with SAS version 9.4 software (SAS Institute, Inc., Cary, NC, USA). All probability values for statistical tests were two-tailed and values of *p* < 0.05 were regarded as statistically significant.

## Results

A total of 352 individuals (306 Palauan and 46 non-Palauan) between 18 and 24 years of age with a mean age of 20.2 years, voluntarily participated in the survey. In total, 37.9% of men and 39.1% of women were found to be currently drinking alcohol and using marijuana. Concurrent alcohol and/or marijuana use were positively correlated (kappa coefficient = 0.2468; *p* < 0.001). Sex-specific characteristics are presented in Table 1. Most of participants were aged 18–20 years, Palauans, and mostly with low education level. The proportion of current smoking was 40.8% in men and 11.8% in women.

**Table 1:** Sex-specific characteristics among 352 individuals between 18 and 24 years of age in Palau, 2013

	Men	Women
	(*n* = 174)	(*n* = 178)
Age		
18–20 years old, %	52.9	67.4
21–24 years old, %	47.1	32.6
Ethnicity		
Palauans, %	86.8	87.1
Non-Palauans, %	13.2	12.9
Education level		
High, %	24.1	25.3
Low, %	75.9	74.7
Current smoking, %	40.8	11.8

As shown in Table 2, 93.1% of men and 73.6% of women had previously consumed alcohol, and 66.7% of men and 36.0% of women had consumed alcohol within 30 days before the survey while 76.4% of men and 52.8% of women had previously used marijuana and 32.8% of men and 19.7% of women had used marijuana within 30 days before the survey.

**Table 2:** Sex-specific proportions of alcohol consumption and marijuana use

	Men	Women	*P* value
	(*n* =174)	(*n* = 178)	
Alcohol consumption			
Ever consumed, %	93.1	73.6	< 0.001***
Consumed alcohol during the past 12 months, %	81.0	57.3	< 0.001***
Frequency of alcohol consumption during the past 12 months, %			0.004**
Daily	1.7	0.6	
5–6 days per week	4.6	0.6	
1–4 days per week	21.3	6.7	
1–3 days per month	32.8	25.3	
Less than once a month	39.7	66.9	
Consumed alcohol during the past 30 days, %	66.7	36.0	< 0.001***
Marijuana use			
Lifetime marijuana use, %			< 0.001***
Non	23.6	47.2	
1 or 2 times	21.3	21.3	
3 or more times	55.1	31.5	
Marijuana use during the past 30 days, %			0.005**
No	67.2	80.3	
Yes	32.8	19.7	

**p < 0.01; ***p < 0.001

**Fig. 1:**
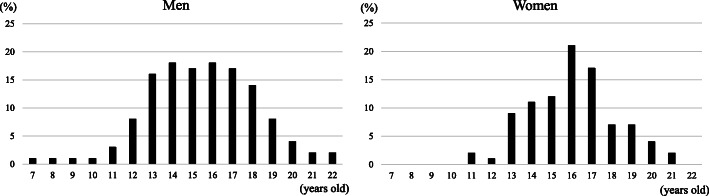
Sex-specific distributions of ages when marijuana was first used

The proportion of current alcohol drinking was higher in the age 21–24 category than the age 18–20 category (73.2% vs. 60.9%, *p* = 0.09 in men and 48.3% vs. 30.0%, *p* = 0.02 in women) as shown in Table 3. The respective proportions among Palauans aged 18–20 years were 90.7% in men and 75.7% in women (data not shown). The proportion of marijuana use did not differ by age groups. As shown in Fig. 1, 37.4 % of men started marijuana at under 14 years old while 24.7% of women started under 14 years old.

**Table 3:** Sex- and age-specific proportions of alcohol consumption and marijuana use

Age groups	Men			Women		
	18–20	21–24	P for difference	18–20	21–24	*P* for difference
	(*n* = 92)	(*n* = 82)		(*n* = 120)	(*n* = 58)	
Alcohol consumption						
Ever consumed, %	90.2	96.3	0.11	70.0	81.0	0.12
Consumed alcohol during the past 12 months, %	78.3	84.1	0.33	51.7	69.0	0.03*
Frequency of alcohol consumption during the past 12 months, %			0.05			0.32
Daily	0	3.7		0.8	0	
5–6 days per week	1.1	8.5		0	1.7	
1–4 days per week	20.7	22.0		5.8	8.6	
1–3 days per month	32.6	32.9		20.0	36.2	
Less than once a month	45.7	32.9		73.3	53.4	
Consumed alcohol during the past 30 days, %	60.9	73.2	0.09	30.0	48.3	0.02*
Marijuana use						
Lifetime marijuana use, %			0.14			0.46
Non	20.7	26.8		48.3	44.3	
1 or 2 times	28.3	13.4		20.0	24.1	
3 or more times	51.1	59.8		31.7	31.0	
Marijuana use during the past 30 days, %			0.71			0.52
No	68.5	65.9		81.7	77.6	
Yes	31.5	34.1		18.3	22.4	

*p< 0.05

In Table 4, the proportion of current drinking was higher in Palauans than the non-Palauans (70.6% vs. 40.6%, *p* = 0.005 in men and 38.8% vs. 16.6%, *p* = 0.04 in women). The proportion of Palauans who had ever used marijuana was higher than in non-Palauans (80.0% vs. 52.9%, *p* = 0.02 in men and 56.1% vs. 30.6%, *p* = 0.09 in women).

**Table 4:** Sex-specific and age-adjusted proportions of alcohol consumption and marijuana use by ethnicity

Ethnicity	Men			Women		
	Palauans	Non-Palauans	P for difference	Palauans	Non-Palauans	P for difference
	(*n* = 151)	(*n* = 23)		(*n* = 155)	(*n* = 23)	
Alcohol consumption						
Ever consumed, %	94.3	85.6	0.13	79.4	34.4	<0.001***
Consumed alcohol during the past 12 months, %	84.2	60.0	0.007**	61.4	29.7	0.004**
Frequency of alcohol consumption during the past 12 months, %			0.37			0.94
Daily	2.2	0		0.6	0	
5–6 days per week	5.5	0		0.7	0	
1–4 days per week	20.5	26.3		6.5	8.6	
1–3 days per month	35.2	16.7		28.5	3.6	
Less than once a month	36.6	59.8		63.7	87.9	
Consumed alcohol during the past 30 days, %	70.6	40.6	0.005**	38.8	16.6	0.04*
Marijuana use						
Lifetime marijuana use, %			0.02*			0.09
Non	20.0	47.1		43.9	69.4	
1 or 2 times	21.4	20.4		23.2	8.9	
3 or more times	58.6	32.5		32.9	21.7	
Marijuana use during the past 30 days, %			0.78			0.88
No	67.0	68.9		80.7	78.2	
Yes	31.1	33.0		21.8	19.3	

*p < 0.05; **p < 0.01; ***p < 0.001

As shown in Table 5, a low education level was associated with increased lifetime marijuana use in men. A similar but non-significant trend was observed in women. Educational levels were not associated with alcohol consumption in either sex. The proportion of men who used marijuana 3 or more times was higher in the low education group than in the high education group (62.5% vs. 32.1%, *p* < 0.001). The corresponding proportion of women tended to be higher in the low education group than in the high education group, but the difference was not statistically significant (33.9% versus 24.4%, *p* = 0.12).

**Table 5:** Sex- specific and age-adjusted proportions of alcohol consumption and marijuana use by education level

Education level	Men			Women		
	High	Low	*P* for difference	High	Low	*P* for difference
	(*n* = 42)	(*n* =132)		(*n* = 45)	(*n* = 133)	
Alcohol consumption						
Ever consumed, %	94.7	92.6	0.65	73.0	73.8	0.91
Consumed alcohol during the past 12 months, %	76.0	82.6	0.35	57.0	57.4	0.96
Frequency of alcohol consumption during the past 12 months, %			0.86			0.51
Daily	1.6	1.8		0	0.7	
5–6 days per week	4.2	4.7		0	0.8	
1–4 days per week	14.1	23.5		2.1	8.3	
1–3 days per month	33.3	32.6		30.3	23.6	
Less than once a month	46.7	37.4		67.7	66.6	
Consumed alcohol during the past 30 days, %	58.4	69.3	0.20	41.4	34.1	0.38
Marijuana use						
Lifetime Marijuana use, %			< 0.001***			0.12
Non	32.7	20.7		55.4	44.4	
1 or 2 times	35.2	16.8		20.3	21.7	
3 or more times	32.1	62.5		24.4	33.9	
Marijuana use during the past 30 days, %			0.30			0.35
No	73.6	65.2		75.4	82.0	
Yes	26.4	34.8		24.6	18.0	

***p < 0.001

## Discussion

We found a high prevalence of alcohol and marijuana use among Palauan men and women aged 18–24 years. Among young adults living in Palau, sex, age, ethnicity, and education level were significant determinants of alcohol or marijuana use. As far as we know, this is the first study to identify demographic and social determinants (sex, age, ethnicity, and education level) of alcohol consumption and marijuana use among Palauans aged 18 to 24 years.

We found that 51.4% (66.7% of men and 36.0% of women) aged 18 to 24 years reported alcohol drinking during the past 30 days. According to the statistics in Palau, one-half of adults were drinkers and the average of 773 cans of standard drinks (beer, wine, and spirits) per adult were imported [8]. The proportions of alcohol drinking during the past 30 days among Americans aged 18–22 years were 51.6% of men and 52.0% of women in 2015 [9]. In England, the proportion who drank alcohol in the last week was 46.9% and 44.5% at ages of 16–24 years in 2013 [10]. The production, sale, and possession of any form of medicinal marijuana products are illegal in Palau; however, we found that 26.3% (32.8% of men and 19.7% of women) aged 18 to 24 years used marijuana during the past 30 days. In the USA, approximately 20% of young adults aged 18 to 25 used marijuana in 2014 [11], indicating that the prevalence of marijuana use was much higher among young adults in Palau. Furthermore, in the present study, many Palauan youth started to use marijuana. The high usage of alcohol and marijuana in Palauan teens was probably due to their easy retail and social access because alcohol was widely sold and marijuana was often cultivated on private farms [12]. The common use of marijuana may also be due to Palauan cultural acceptance. In 1987, Evans reported from Palau that “It was clear that marijuana was not perceived to be a problem substance by users or law enforcement officers, and marijuana was grown openly in Palau” [13].

The Youth Risk Behavior Surveillance System (YRBSS) for Pacific Island countries found that the proportion of current student drinkers aged 13 to 15 years varied between 10.3% and 43.7% in boys and 5.1% and 28.7% in girls [14]. The Global School-Based Health Survey (GSHS) reported similar proportions of current drinkers: 40.6% in boys and 14.1% in girls of grades 6 to 10 in four Pacific Island countries [15]. For young adults aged 25 to 64 years, the age-standardized proportions of current drinkers varied more among Pacific Island countries according to the WHO STEPwise approach to surveillance (STEPS): 13.1% to 96.0% in men and 1.2% to 90.1% in women [14]. In the present study, we found that the proportion of alcohol drinkers in the 30-day period prior to the survey was 66.7% in men and 36.0% in women, indicating that habitual alcohol consumption is very common among Palauan adolescents and young adults.

The proportions of those Palauans who had used marijuana before were 68.6% in boys and 56.9% in girls of grades 9 to 12 according to the 2013 YRBSS [16]. We found that the proportion of prior marijuana users was 76.4% in men and 52.8% in women aged 18 to 24 years. Therefore, marijuana usage among men may become common after students enter college due to peer pressure and easy access.

In the present study, among men, low education was associated with lifetime marijuana use but not with alcohol consumption. According to systematic reviews, childhood social disadvantages, including low education, were associated with risk of cannabis use (but not alcohol use) from adolescence to adulthood [17, 18]. However, the higher proportion of lifetime marijuana use in the low education group does not necessarily imply that low education per se led to marijuana use since it may be strongly confounded by low family income. Leniency among low-income and low education parents may allow more teenagers or young adults to start marijuana usage [19]. Among women in the preset study, low education tended to be associated with lifetime marijuana use, but the association did not reach statistical significance. A study of 45 men and 48 women aged 8–30 years showed that the magnitude of risk behaviors evaluated by gambling task were higher in men than in women for all age groups [20]. The tendency of taking non-risky behaviors in women could make the association between education and lifetime marijuana use weaker.

The strength of this study is in its clarification of demographic and social determinants (sex, age, ethnicity, and education levels) of alcohol consumption and marijuana use among young adult Palauans. However, there are several limitations in the present study. First, the participation rate of non-PCC students was not high (25%) and approximately 80% of the participants lived in Koror. However, we sampled over 20% of the total 18 to 24-year-old population living in Palau, of which more than 80% resided in Koror [7]. Therefore, the impact of selection bias was likely to be negligible. Second, we did not obtain detailed information regarding the amounts and usage patterns of alcoholic beverages and marijuana. Third, the law governing participants’ nation may influence on the present results; however, we cannot evaluate that possibility because the detailed information on nationality among non-Palauans was not obtained.

## Conclusion

Sex, age, ethnicity, and education were significant determinants of alcohol or marijuana use among young adults living in Palau, particularly Palauans. This study was executed to serve as a baseline reference for the further development of public health measures to control alcohol and drug use in Palau.

## Data Availability

The datasets generated during and/or analyzed during the current study are not publicly available due to ethical consideration.
